# Supplementary material for: Erector spinae plane block versus paravertebral block on postoperative quality of recovery in obese patients undergoing laparoscopic sleeve gastrectomy: a randomized controlled trial
Source: PeerJ. 2024 May 28;12:e17431. doi: 10.7717/peerj.17431 (PMC11141559; doi:10.7717/peerj.17431)
Supplement: Supplemental Information 3 [file peerj-12-17431-s003.docx]

**Project summary**

To investigate the effect of erector spinae plane block (ESPB) vs. paravertebral blocks (PVB) on postoperative quality of recovery (QoR) in patients undergoing laparoscopic sleeve gastrectomy (LSG). One hundred and ten patients undergoing elective LSG under general anesthesia were randomly allocated to receive ultrasound-guided bilateral ESPB or PVB at T8 levels with 40mL 0.33% ropivacaine before anesthesia induction. The primary outcome was the postoperative QoR-15 score at 24h. Secondary outcomes included postoperative QoR-15 score at 48h, numeric rating scales (NRS) score at rest and during coughing at 0.5, 2, 6, 12, 24, 36, and 48h after surgery, the postoperative time to first mobilization out of bed, time to first pass flatus, postoperative cumulative oxycodone consumption and the incidence of postoperative nausea and vomiting (PONV).

**General information**

**Erector spinae plane block versus paravertebral block on postoperative quality of recovery in obese patients undergoing laparoscopic sleeve gastrectomy: A randomized controlled trial**

Guanyu Yang^1^, Pengfei Wang^1^, Yue Yin^1^, Huan Qu^1^, Xin Zhao^1^, Xiaogao Jin^1^, Qinjun Chu^1^

^1^Department of Anesthesiology and Perioperative Medicine, Zhengzhou Central Hospital Affiliated to Zhengzhou University, Zhengzhou, Henan, China

Corresponding Author:

Qinjun Chu^1^

Tongbai North Road 16, Zhengzhou, Henan, 450007, China

Email address: 17888551304@163.com

**Rationale & background information**

Obesity has been one of the most common medical conditions in developed countries^1^, and this disorder is associated with high incidences of hypertension, dyslipidaemia, cardiovascular disease, type 2 diabetes mellitus^2^. Surgical approaches may be the only effective long-term treatment for obesity^3^, laparoscopic sleeve gastrectomy (LSG) is a popular surgical procedure for bariatric surgery^4^. Most patients undergoing bariatric surgery experienced moderate to severe acute pain after surgery, adequate postoperative analgesia is crucial to enhance the recovery of patients^5^. Multimodal analgesia, including regional analgesia has become a standard protocol for acute pain management. In recent years, there has been an increase in the use of regional block in multimodal analgesia.

The analgesic effects of erector spinae plane block (ESPB) and paravertebral block (PVB) have been fully confirmed in postoperative analgesia of thoracic surgery^6-9^, but there are few studies on the analgesic effects of these two methods after upper abdominal surgery, especially in patients with obesity.

Therefore, we conducted this prospective randomized controlled trial to compare the effects of two analgesic methods on postoperative quality of recovery (QoR) in patients undergoing LSG.

**References**

1. Smith KB, Smith MS. Obesity Statistics. Prim Care. 2016;43:121-ix.

2. Varban OA, Cassidy RB, Bonham A, Carlin AM, Ghaferi A, Finks JF; Michigan Bariatric Surgery Collaborative. Factors Associated With Achieving a Body Mass Index of Less Than 30 After Bariatric Surgery. JAMA Surg. 2017;152:1058-1064.

3. Glass J, Chaudhry A, Zeeshan MS, Ramzan Z. New Era: Endoscopic treatment options in obesity-a paradigm shift. World J Gastroenterol. 2019;25:4567-4579.

4. Alharbi SR. Computed Tomography-based Diagnosis of Post-laparoscopic Sleeve Gastrectomy Gastric Leak. J Clin Imaging Sci. 2020;10:8.

5. Nimmo SM, Foo ITH, Paterson HM. Enhanced recovery after surgery: Pain management. J Surg Oncol. 2017;116: 583-591.

6. Yao Y, Fu S, Dai S, Yun J, Zeng M, Li H, et al. Impact of ultrasound-guided erector spinae plane block on postoperative quality of recovery in video-assisted thoracic surgery: A prospective, randomized, controlled trial. J Clin Anesth. 2020;63:109783.

7. Taketa Y, Irisawa Y, Fujitani T. Comparison of ultrasound-guided erector spinae plane block and thoracic paravertebral block for postoperative analgesia after video-assisted thoracic surgery: a randomized controlled non-inferiority clinical trial. Reg Anesth Pain Med. 2019; rapm-2019-100827.

8. Koo CH, Lee HT, Na HS, Ryu JH, Shin HJ. Efficacy of Erector Spinae Plane Block for Analgesia in Thoracic Surgery: A Systematic Review and Meta-Analysis. J Cardiothorac Vasc Anesth. 2022;36:1387-1395.

9. D'Ercole F, Arora H, Kumar PA. Paravertebral Block for Thoracic Surgery. J Cardiothorac Vasc Anesth. 2018;32:915-927.

**Study goals and objectives**

This study intends to investigate the effect of erector spinae plane block vs. paravertebral blocks on postoperative quality of recovery in patients undergoing laparoscopic sleeve gastrectomy.

**Study design**

This is a single-center, randomized, controlled trial. Patients undergoing LSG in our hospital between February, 2021 and June, 2021 were recruited for this study. All patients signed informed consent before the start of the trial. The inclusion criteria were as follows: 1) Patients aged 18-45 years; 2) 30kg/m^2^≤body mass index (BMI)≤40kg/m^2^ ; 3) American Society of Anesthesiologists (ASA) physical status II or III. Exclusion criteria were: 1) blood system diseases or blood coagulation dysfunction; 2) infection at the puncture site; 3) allergy to local anesthetics or NSAIDs; 4) psychiatric history or neurological diseases; 5) inability to communicate.

**Methodology**

Patients were randomly allocated into the ultrasound-guided ESPB group (Group ESPB) or ultrasound-guided PVB group (Group PVB) at T8 levels using random numbers generated by Excel. The investigators prepared sealed, opaque, sequentially numbered envelopes containing cards with assignment information based on the list. Upon arrival of eligible patients in the operating room, the investigator nurse assigned participants by opening the envelopes containing the pre-written assignments. Patients, surgeons, post-anesthesia care unit (PACU) staff, data collectors, and those performing statistical analyses were blinded to the grouping throughout the observation period, including all post-operative follow-up periods. All patients received general anesthesia after the regional blocks were performed by the same anesthesiologist with more than 3 years of experience in nerve blocks.

Upon admission to the operating room, an intravenous (IV) line was established with an 18-gauge IV cannula on the forearm. Ringer’s lactate (RL) solution at a rate of 2-4 ml/kg/h was used to maintain the basal fluid. Rescue vasoactive agents including atropine, ephedrine or epinephrine were available at the bedside. All patients were placed in the lateral supine position for regional block in two groups after receiving 5-10μg sufentanil intravenously.

Ultrasound-guided ESPB: Using ultrasonic sliding down from the C7 spinous process until identifying T8 spinous process. Then, a 2-5 MHz convex array transducer (HFL38xi, FUJIFILM SonoSite, USA) was located longitudinally 2-3 cm lateral to the T8 transverse process. After identifying the trapezius and erector spinae muscles, a 24-gauge needle was inserted from the caudal side to the cephalic side using an in-plane technique. Then confirming that the tip is located on the deep surface of the erector spinae muscle and the surface of the transverse process, hydrodissection was performed on the interfascial plane with 3mL saline solution, followed by an injection of 0.33% ropivacaine 20mL. The same procedure was applied to the opposite side ESPB.

Ultrasound-guided PVB: The T8 transverse process was identified using the same method as ultrasound-guided ESPB. After identifying the pleura, a 24-gauge needle was inserted out-plane and directed to the T8 transverse process. Once the transverse process was contacted, move the needle off the bone in a caudal direction to penetrate the superior costotransverse ligament. Blood vessel or pleural injection negative was confirmed by aspiration, then the local anesthetic delivery would be performed, and the pleura would be pushed down. Patients in group PVB received the same amount of ropivacaine through T8 paravertebral spaces. The same procedure was applied to the opposite side PVB.

Propofol 1.5-2.5mg/kg, sufentanil 0.5μg/kg, rocuronium 0.6-1.2mg/kg were intravenously administered as anesthetic induction based on the patient's lean body weight (LBW). Mechanical ventilation was initiated after endotracheal intubation. Anesthesia was maintained with propofol (50-100 μg.kg^-1^.min^-1^), remifentanil (0.05-0.25 μg.kg^-1^.min^-1^) and intermittent bolus injection of rocuronium. The BIS value was maintained within a range of 40-60. Intraoperative blood pressure fluctuation was maintained at ±20% of baseline level.

Flurbiprofen (100mg) was administered intravenously half an hour before completion of surgery. After surgery, all patients were extubated after intravenous injection of sugammadex (Bridion, Merck Sharp Dohme, New Jersey, USA) at a dose of 4 mg/kg and taken to the PACU. All patients were evaluated by the anesthesiologist in PACU and returned to the ward by the nurse anesthetist after meeting the criteria for leaving PACU. All patients received PCIA pump (REHN11, Renxian Medical Corporation, Jiangsu, China) for postoperative analgesia with the same protocol before leaving the operating room in two groups. The PCIA was flurbiprofen 200mg plus oxycodone 20mg, diluted to 100mL with normal saline. The parameter was set as the background free infusion dose, the single dose was 5mL, the interval was 5min, and the limited dose was 20mL.

**Safety considerations**

Sign informed consent with each patient. Inform of possible risks and measures to be taken. Patients can opt-out at any time. The patient's vital signs were kept stable during the operation.

**Follow-up**

Follow-up time for each patient will be 48 hours postoperatively.

**Data management and statistical analysis**

Sample size was determined using Gpower 3.1. Based on our preliminary study, the QoR-15 score at 24 hours after preoperative PVB at T8 was 122±12.1 for patients undergoing LSG, while the QoR-15 score at 24 hours after preoperative ESPB at T8 was 129±10.8. Assuming α=0.05, 1-β=0.8, a two-tailed test was conducted, resulting in a calculated sample size of 44 for each group. To account for a potential 20% dropout rate, a total of 110 participants were enrolled in this study.

R software was utilized to analyze all statistical data. Mean ± standard deviation was used to express measurement data with a normal distribution, and a two independent samples t-test was employed to compare the means between the two groups. For measurement data with a non-normal distribution, the median (interquartile range) was used, and the Mann-Whitney U test was used for between-group comparisons. Categorical variables were compared using the chi-squared test or Fisher's exact test. A significance level of P<0.05 was considered statistically significant.

**Quality assurance**

Studies are carried out in strict accordance with inclusion and exclusion criteria. All surgeries will be performed by the same team of surgeons. Try to use objective indicators to evaluate the research results.

**Expected outcomes of the study**

We predicted that the quality of recovery at 24h and 48h was the same in the two groups.

**Dissemination of results and publication policy**

It is hoped that our results will be published and made available to other centers.

**Duration of the project**

The study is expected to start enrolling patients on February 2021, with an expectation of 150 days. The follow-up time will be 48h after surgery for each patient.

**Problems anticipated**

Withdrawal of the patient and the refusal to participate in follow-up may occur during the trial. These patients will not be included in the final statistical analysis.

**Project management**

Guanyu Yang: Conceptualization, Investigation, Methodology, Formal analysis, Writing-original draft. Pengfei Wang: Investigation, Project administration. Yue Yin: Investigation, Project administration. Huan Qu: Investigation, Project administration. Xin Zhao: Investigation, Project administration. Xiaogao Jin: Conceptualization, Formal analysis, Project administration, Supervision, Writing - original draft. Qinjun Chu: Conceptualization, Investigation, Formal analysis, Resources, Supervision, Project administration, Writing - original draft.

**Ethics**

The trial protocol will be explained in detail to each patient and informed of what they would participate in, of the possible damage and corresponding measures, eventually enabling each participating patient to sign an informed consent form. This study has been approved by the hospital ethics committee.

**Budget**

The expected cost is mainly the published APC as well as the labor fee of the participants.

**Other support for the project**

This research did not receive any specific grant from funding agencies in the public, commercial, or not-for-profit sectors.
